# Decline in Female Sexual Function From Late Pregnancy to Early Postpartum: A Prospective Longitudinal Study in Nicaragua

**DOI:** 10.7759/cureus.110490

**Published:** 2026-06-08

**Authors:** María Esther Suárez García, Karen Vanessa Herrera, Christopher Kaleb Romero Ríos, Erick Alexander de Jesús Chamorro Segovia, Marcela Conrado Chamorro, Andres Rivera, Emilio J Davila Alvarez

**Affiliations:** 1 Obstetrics and Gynecology, Hospital Militar Escuela “Dr. Alejandro Dávila Bolaños”, Managua, NIC; 2 Centro de Investigaciones y Estudios de la Salud, Universidad Nacional Autónoma de Nicaragua, Managua, NIC; 3 School of Medicine, Hospital Militar Escuela "Dr. Alejandro Dávila Bolaños", Managua, NIC; 4 Obstetrics and Gynecology, Hospital Militar Escuela "Dr. Alejandro Dávila Bolaños", Managua, NIC

**Keywords:** cesarean section, female sexual function, fsfi, longitudinal study, maternal health, postpartum period, pregnancy, reproductive health, sexual dysfunction, sexual health

## Abstract

Background: Sexual function is a key component of maternal well-being and is frequently affected during pregnancy and the postpartum period due to hormonal, physical, and psychosocial changes. Despite its clinical relevance, postpartum sexual dysfunction remains underrecognized in routine care.

Objective: To evaluate changes in female sexual function between the third trimester of pregnancy and the early postpartum period and to identify factors associated with postpartum sexual dysfunction.

Methods: A prospective longitudinal observational study was conducted on 184 women attending the Department of Gynecology and Obstetrics of a tertiary care center in Nicaragua. Participants were assessed during the third trimester of pregnancy and again at 4-8 weeks postpartum (mean: six weeks) using the Female Sexual Function Index (FSFI). Descriptive statistics were calculated, paired comparisons were performed using Student's t-test, and normality of differences was assessed with the Shapiro-Wilk test. A multivariable Poisson regression model with robust variance was constructed to identify factors independently associated with postpartum sexual dysfunction, estimating adjusted prevalence ratios (aPR).

Results: The mean age was 29.6 ± 5.2 years. Mode of delivery was predominantly vaginal (n = 110; 59.8%), with cesarean section accounting for 40.2% (n = 74) of cases. During pregnancy, sexual function was largely preserved (mean FSFI total score: 27.81 ± 2.96, above the clinical threshold of 26.55). In contrast, a marked decline was observed postpartum (mean FSFI total score: 16.64 ± 4.76; p < 0.0001). When the validated cutoff of FSFI < 26.55 was applied, 100% of postpartum participants met criteria for sexual dysfunction. Domain-level analysis showed statistically significant reductions in all six FSFI domains, with the greatest decreases in lubrication (−2.06 points), arousal (−1.71), and desire (−1.69). The prevalence of postpartum sexual dysfunction based on the analytic binary variable was 62.0% (114/184). In the multivariable analysis, university education (aPR = 1.84) and multiparity (aPR = 1.20) were associated with sexual dysfunction. The presence of anxiety or sadness showed the strongest association, increasing the prevalence fivefold (aPR = 5.06). Participation in institutional support groups was associated with a 37% reduction in dysfunction prevalence (aPR = 0.63). Mode of delivery was not significantly associated with postpartum sexual dysfunction (aPR = 1.055; p = 0.479).

Conclusions: Female sexual function declines significantly in the early postpartum period, affecting all domains of sexual response. These findings highlight the urgent need for integrated, biopsychosocial approaches to postpartum care, particularly in resource-limited settings where this dimension of maternal health remains systematically overlooked.

## Introduction

Female sexual function is a multidimensional construct that includes desire, arousal, lubrication, orgasm, satisfaction, and pain and is shaped by the interaction of biological, psychological, and relational factors [1]. Pregnancy and the postpartum period are particularly sensitive stages in this regard because they involve profound hormonal, anatomical, and psychosocial changes that may alter sexual well-being. Current evidence indicates that sexual dysfunction during pregnancy is highly prevalent, with pooled estimates approaching 70% globally and even higher frequencies reported in the third trimester [2]. The postpartum period appears to be even more vulnerable, especially during the first months after childbirth, when reduced desire, impaired lubrication, sexual pain, and overall sexual dysfunction are commonly reported [3,4].

Although postpartum sexual dysfunction is common and clinically relevant, it remains insufficiently recognized in routine maternal care. Many women report unmet informational needs and limited counseling regarding sexual health after childbirth, despite the substantial impact of these symptoms on quality of life, intimate relationships, and psychological well-being [5,6]. Cultural paradigms often discourage women from openly discussing their sexuality or seeking clinical help; this is exacerbated by a systemic deficiency in educational programming from the onset of gestation. Longitudinal studies suggest that sexual function typically worsens from late pregnancy to the early postpartum period and then gradually recovers over time, although this trajectory is heterogeneous and appears to be influenced not only by biological and obstetric factors but also by depressive symptoms, relationship quality, body image concerns, and other psychosocial determinants [1,7]. However, evidence from low- and middle-income settings remains limited, particularly regarding longitudinal data and the influence of contextual factors such as access to counseling and emotional vulnerability in clinical populations. This gap is especially relevant in Central America, where postpartum sexual health remains insufficiently contextualized within maternal care frameworks. In this context, the primary aim of this prospective longitudinal study was to evaluate the trajectory of female sexual function from the third trimester of pregnancy to the early postpartum period in a Nicaraguan cohort. Our secondary objective was to identify sociodemographic, obstetric, and psychosocial factors independently associated with early postpartum sexual dysfunction. We hypothesized that sexual function scores would decline significantly across all domains postpartum and that psychosocial variables, rather than obstetric factors such as mode of delivery, would be the primary factors associated with this decline.

## Materials and methods

Study design and setting

A prospective, longitudinal, analytical observational study was conducted between May and October 2025 at the Department of Gynecology and Obstetrics of the Hospital Militar Escuela "Dr. Alejandro Dávila Bolaños," a tertiary care referral center in Managua, Nicaragua. The study was designed and reported following the Strengthening the Reporting of Observational Studies in Epidemiology (STROBE) guidelines [8].

Participants and sampling

The study enrolled pregnant women in their third trimester who subsequently completed their pregnancy and attended a follow-up assessment 4 to 8 weeks postpartum (mean: six weeks). The source population comprised the 350 women attending the Department of Gynecology and Obstetrics during the study period who met these criteria. Additional inclusion criteria required participants to have been sexually active during the third trimester of pregnancy, to have resumed sexual activity between 4 and 8 weeks postpartum, to provide voluntary informed consent, and to possess the literacy or cognitive ability necessary to complete the research questionnaires.

Exclusion criteria included severe obstetric complications preventing postpartum evaluation, previously diagnosed psychiatric disorders interfering with questionnaire responses, incomplete follow-up, or withdrawal from the study at any stage.

A non-probabilistic convenience sampling approach was used due to feasibility constraints within the clinical setting; however, all eligible participants during the study period were consecutively invited to minimize selection bias.

Sample size

The sample size was estimated using OpenEpi software, based on a finite population of 350 individuals (the total number of eligible women attending the service during the study period). Utilizing a 95% confidence level, an expected proportion of 50%, and a 5% margin of error, the minimum required sample was calculated at 184 participants. The final sample successfully met this target.

Data collection and measures

Female sexual function was quantified using the Female Sexual Function Index (FSFI), a validated, multidimensional self-report instrument encompassing 19 items across six distinct domains: desire, arousal, lubrication, orgasm, satisfaction, and pain [9]. Domain-specific scores are weighted and aggregated to yield a total score ranging from 2 to 36, where higher values reflect superior sexual function. Participants completed the questionnaires privately in a confidential setting. The principal investigator provided standardized instructions and remained available to clarify procedural questions if needed, without influencing responses. To ensure quality control and minimize information bias, follow-up assessments at the 4-8 week postpartum visit were conducted following a standardized protocol, with trained personnel available to ensure the completeness of the data.

The primary endpoint was the total FSFI score assessed longitudinally across two time points: the third trimester of pregnancy and the early postpartum period. Secondary outcomes included individual domain scores and the prevalence of postpartum sexual dysfunction, defined by a validated clinical threshold of total FSFI score below 26.55 [10]. Baseline covariates comprised sociodemographic data (age, educational attainment, marital status), obstetric history (parity, mode of delivery), and psychosocial variables, including anxiety or depressive symptoms, perceived partner support, body image disturbances, and prior receipt of sexual health counseling.

Psychosocial variables were collected through structured self-report questions. Emotional symptoms were assessed using a dichotomous self-reported variable referring to the presence of anxiety and/or sadness during pregnancy or the postpartum period. Body image concerns were evaluated through participant self-perception regarding the impact of postpartum body changes on sexual desire and self-esteem. Participation in institutional support groups and prior receipt of sexual health counseling were also recorded as binary variables (yes/no). These variables were exploratory in nature and were not based on validated psychometric scales.

In addition to the FSFI, participants were asked a direct self-perception question regarding postpartum sexual dysfunction: 'Do you consider that you have experienced sexual difficulties or dysfunction during the postpartum period?' Responses were recorded as a dichotomous yes/no variable.

Statistical analysis

Data were analyzed using IBM Corp. Released 2021. IBM SPSS Statistics for Windows, Version 27. Armonk, NY: IBM Corp. Continuous variables were summarized as mean ± standard deviation, and categorical variables as frequencies and percentages.

Comparisons between FSFI scores during pregnancy and postpartum were performed using paired Student's t-tests, given the repeated measures design and continuous nature of the variables. Prior to inferential testing, normality of paired differences was assessed using the Shapiro-Wilk test. A two-sided p-value < 0.05 was considered statistically significant.

To identify factors independently associated with postpartum sexual dysfunction, a multivariable Poisson regression model with robust variance (sandwich estimator) was constructed. This approach was selected over traditional logistic regression to avoid the overestimation of risk commonly observed with odds ratios when the outcome is highly frequent. Because 100% of the postpartum sample scored below the validated FSFI clinical cutoff of < 26.55, utilizing this threshold resulted in zero outcome variance, which precludes stable multivariable model estimation. Consequently, to allow for the mathematical identification of associated risk factors, the prospectively collected, self-reported dichotomous variable for sexual dysfunction was utilized as the dependent variable. Mode of delivery (vaginal vs. cesarean section) was additionally included as a covariate in the model. Adjusted prevalence ratios (aPRs) with 95% confidence intervals were reported. Model stability was assessed by evaluating coefficient behavior and avoiding overfitting, considering the number of outcome events relative to predictors.

Ethical considerations

The study was conducted in accordance with the Declaration of Helsinki [11] and was approved by the Institutional Ethics Committee of the Hospital Militar Escuela "Dr. Alejandro Dávila Bolaños" with approval code (HMEADB-CE-2025-04). All participants provided written informed consent prior to enrollment. Confidentiality and anonymity were ensured through coded data and restricted access to study information. The study involved minimal risk, as it was based exclusively on questionnaire data collection.

## Results

Participant characteristics

A total of 184 women were included in the final analysis, all of whom completed both study assessments. The mean age was 29.6 ± 5.2 years. Most participants were in a stable relationship (81.0%), either married or cohabiting, while 19.0% were single. More than half of the sample had attained university-level education (59.2%). The obstetric history revealed a predominance of multiparity, with women in their third pregnancy comprising 55.4% of the study population. Mode of delivery was predominantly vaginal (n = 110; 59.8%), with cesarean section accounting for 40.2% (n = 74) of cases. The higher proportion of vaginal deliveries likely reflects the inclusion criterion requiring resumption of sexual activity within 4-8 weeks postpartum, as cesarean recovery may be associated with delayed sexual resumption in some women. Baseline characteristics are summarized in Table 1.

**Table 1:** Baseline sociodemographic and obstetric characteristics of the study population (N = 184) † For regression analysis, parity was dichotomized as primiparous (1st pregnancy) vs. multiparous (2nd pregnancy or more).

Characteristics	n (%)
Age (years)	
20–24	44 (23.9)
25–29	58 (31.5)
30–34	38 (20.7)
35–39	37 (20.1)
≥40	7 (3.8)
Marital Status	
Married	75 (40.8)
Cohabiting	74 (40.2)
Single	35 (19.0)
Educational Level	
Primary education	68 (37.0)
Secondary education	7 (3.8)
University degree	109 (59.2)
Parity†	
Primiparous (1st pregnancy)	18 (9.8)
Multiparous (2nd pregnancy)	43 (23.4)
Multiparous (3rd pregnancy)	102 (55.4)
Multiparous (≥4th pregnancy)	21 (11.4)
Mode of delivery	
Cesarean section	74 (40.2)
Vaginal delivery	110 (59.8)

Sexual function during the third trimester of pregnancy

During the third trimester, overall sexual function was largely preserved, with a mean FSFI total score of 27.81 ± 2.96, above the clinical threshold for dysfunction (26.55). Analysis of individual FSFI domains revealed that the majority of participants maintained moderate to high functional levels. Robust sexual desire was reported by 75.0% of the cohort, while a substantial proportion (86.9%) demonstrated elevated arousal. Physiological markers remained favorable, with adequate vaginal lubrication reported by 76.6% of respondents. Orgasmic function was preserved in the vast majority of the sample (97.8%), and high levels of sexual satisfaction were observed in 88.0% of participants. Sexual pain was characterized as predominantly mild, with a notable absence of severe symptomatology. Detailed domain-specific distributions are presented in Table 2.

**Table 2:** Distribution of Female Sexual Function Index (FSFI) domains during the third trimester of pregnancy and the early postpartum period (N = 184) * 15 women (8.2%) reported new-onset high-frequency pain postpartum, a category entirely absent during pregnancy.

FSFI Domain	Pregnancy n (%)	Postpartum n (%)
Sexual Desire		
None	2 (1.1)	19 (10.3)
Low	3 (1.6)	111 (60.3)
Moderate	41 (22.3)	54 (29.3)
High	107 (58.2)	0 (0.0)
Very High	31 (16.8)	0 (0.0)
Sexual Arousal		
None	2 (1.1)	0 (0.0)
Low	3 (1.6)	130 (70.7)
Moderate	19 (10.3)	54 (29.3)
High	129 (70.1)	0 (0.0)
Very High	31 (16.8)	0 (0.0)
Vaginal Lubrication		
None	2 (1.1)	114 (62.0)
Low	3 (1.6)	16 (8.7)
Moderate	38 (20.7)	54 (29.3)
High	141 (76.6)	0 (0.0)
Orgasm		
None	0 (0.0)	111 (60.3)
Low	3 (1.6)	18 (9.8)
Moderate	149 (81.0)	55 (29.9)
High	31 (16.8)	0 (0.0)
Overall Satisfaction		
None	0 (0.0)	4 (2.2)
Low	3 (1.6)	125 (67.9)
Moderate	19 (10.3)	55 (29.9)
High	162 (88.0)	0 (0.0)
Pain		
None	0 (0.0)	52 (28.3)
Low frequency	34 (18.5)	18 (9.8)
Moderate frequency	150 (81.5)	99 (53.8)
High frequency*	0 (0.0)	15 (8.2)

Sexual function during the postpartum period

All 184 participants included in the postpartum assessment had resumed sexual activity within the 4-8-week postpartum window, as this constituted an inclusion criterion of the study. The FSFI was therefore administered exclusively to sexually active women in the early puerperium.

When the validated FSFI clinical cutoff of < 26.55 was applied, 100% of postpartum participants (184/184) met criteria for sexual dysfunction, confirming a generalized and severe decline in sexual function during the early postpartum period. Given that a 100% outcome prevalence precludes adequate variability for multivariable regression modeling, the binary outcome used in the Poisson regression was based on a self-reported sexual dysfunction variable recorded in the analytic database. The prevalence of postpartum sexual dysfunction based on this variable was 62.0% (114/184 women), with the remaining 38.0% (70/184) classified as not having sexual dysfunction.

The postpartum period showed a pronounced decline in sexual function, with a mean FSFI total score of 16.64 ± 4.76, well below the clinical threshold. Low or absent sexual desire affected 70.6% of participants, with a comparable proportion reporting reduced sexual arousal (70.7%). Vaginal lubrication was the most compromised domain, with 70.7% of women experiencing low or absent lubrication. Orgasmic dysfunction was also common, affecting 70.1% of participants, and sexual satisfaction decreased substantially, with 67.9% reporting low levels. The pain domain also showed significant deterioration: 53.8% of women reported moderate-frequency pain and 8.2% reported high-frequency pain postpartum, a category that was absent during pregnancy. Domain-specific postpartum findings are summarized in Table 2.

Comparison between pregnancy and postpartum

Prior to inferential testing, the Shapiro-Wilk test indicated significant deviation from normality in the paired differences for all domains and the total FSFI score (total: W = 0.784, p < 0.001; desire: W = 0.512, p < 0.001; arousal: W = 0.559, p < 0.001; lubrication: W = 0.793, p < 0.001; orgasm: W = 0.762, p < 0.001; satisfaction: W = 0.664, p < 0.001; pain: W = 0.785, p < 0.001). 

Comparative analysis revealed statistically significant reductions in both the total FSFI score and all six individual domains between the third trimester and the postpartum period. The mean total FSFI score decreased from 27.81 ± 2.96 during pregnancy to 16.64 ± 4.76 postpartum, a decline of 11.17 points (t(183) = −32.54; p < 0.0001), indicating a clinically meaningful deterioration in overall sexual function.

**Figure 1:**
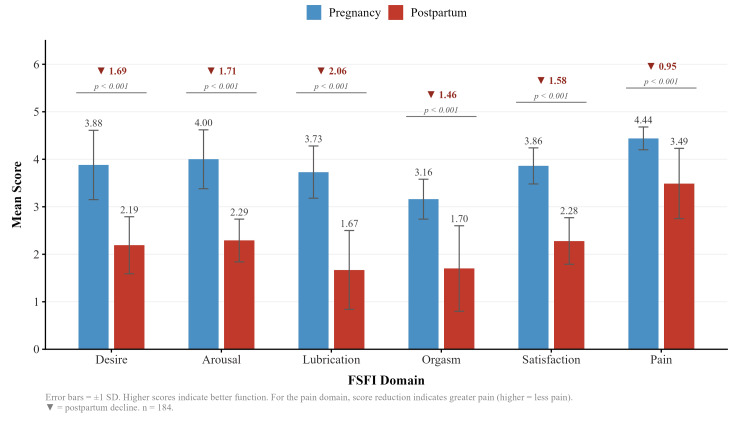
Decline in FSFI domain scores from late pregnancy to early postpartum A clustered bar chart comparing mean FSFI domain scores between the third trimester of pregnancy and the early postpartum period (4–8 weeks; mean six weeks). Blue bars represent pregnancy scores; red bars represent postpartum scores. Error bars indicate ±1 standard deviation. All six domains showed statistically significant deterioration postpartum. For the pain domain, the reduction in score indicates greater pain, since on the FSFI scale higher values correspond to less pain. All comparisons p < 0.001 (paired Student’s t-test). n = 184

At the domain level, the greatest reduction occurred in lubrication (mean difference: −2.06; t(183) = −26.19; p < 0.001), followed by arousal (−1.71; t(183) = −32.38; p < 0.001) and desire (−1.69; t(183) = −33.19; p < 0.001). Significant decreases were also observed in satisfaction (−1.58; t(183) = −41.99; p < 0.001), orgasm (−1.46; t(183) = −26.36; p < 0.001), and pain (−0.95; t(183) = −15.37; p < 0.001). The reduction in the pain domain score reflects greater pain during the postpartum period, since on the FSFI scale, higher values correspond to less pain. These findings are presented in Table 3 and Figure 1.

**Table 3:** Comparison of FSFI domain scores between pregnancy and postpartum (N = 184) ‡ On the FSFI scale, higher scores in the pain domain indicate less pain. The negative difference (−0.95) therefore indicates greater pain during sexual activity in the postpartum period compared to pregnancy.

FSFI Domain	Pregnancy Mean±SD	Postpartum Mean±SD	Difference	Paired t	P-value
Desire	3.88±0.73	2.19±0.60	−1.69	−33.19	<0.001
Arousal	4.00±0.62	2.29±0.45	−1.71	−32.38	<0.001
Lubrication	3.73±0.55	1.67±0.83	−2.06	−26.19	<0.001
Orgasm	3.16±0.42	1.70±0.90	−1.46	−26.36	<0.001
Satisfaction	3.86±0.38	2.28±0.49	−1.58	−41.99	<0.001
Pain‡	4.44±0.24	3.49±0.74	−0.95	−15.37	<0.001
Total FSFI†	27.81±2.96	16.64±4.76	−11.17	−32.54	<0.001

Factors associated with postpartum sexual dysfunction

A multivariable Poisson regression with robust variance estimated the adjusted prevalence ratios of factors associated with sexual dysfunction at 4-8 weeks postpartum among the 184 women included.

Women with a university education showed a higher prevalence of sexual dysfunction compared to those without this level of education (aPR = 1.84; 95% CI: 1.43-2.36; p < 0.001). Multiparous women had a 20% higher prevalence of sexual dysfunction compared to primiparous women (aPR = 1.20; 95% CI: 1.01-1.44; p = 0.038); although statistically significant, the effect size was modest, and the confidence interval was close to the null value.

**Figure 2:**
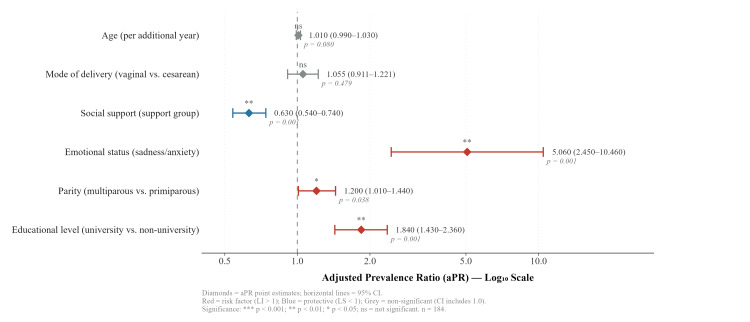
Factors associated with early postpartum sexual dysfunction Adjusted Prevalence Ratios from Multivariable Poisson Regression. Forest plot displaying adjusted prevalence ratios (aPR) with 95% confidence intervals (CIs) from a multivariable Poisson regression model with robust variance. Diamond shapes represent point estimates; horizontal lines represent 95% CI. The vertical dashed line at aPR = 1.0 denotes the null effect. Red diamonds indicate statistically significant risk factors; blue diamond indicates a statistically significant protective association (causal inference is limited by observational design); grey diamond indicates a non-significant association. Significance levels: *** p < 0.001; ** p < 0.01; * p < 0.05; ns = not significant. The x-axis is displayed on a log₁₀ scale. n = 184.

With respect to psychoemotional factors, the presence of sadness or anxiety during pregnancy or the postpartum period showed the strongest association, increasing the prevalence of sexual dysfunction by more than fivefold (aPR = 5.06; 95% CI: 2.45-10.46; p < 0.001). Conversely, participation in institutional support groups was associated with a 37% reduction in the prevalence of sexual dysfunction (aPR = 0.63; 95% CI: 0.54-0.74; p < 0.001). Maternal age was not significantly associated with sexual dysfunction (aPR = 1.01; 95% CI: 0.99-1.03; p = 0.080). Mode of delivery was not significantly associated with postpartum sexual dysfunction after multivariable adjustment (aPR = 1.055; 95% CI: 0.911-1.221; p = 0.479). The results are presented in Figure 2.

## Discussion

This prospective longitudinal study documented a substantial and clinically meaningful decline in female sexual function between the third trimester of pregnancy and the early postpartum period in a Nicaraguan tertiary care population. The assessment at 4-8 weeks postpartum is clinically pertinent, as this interval corresponds to the period during which the reproductive organs complete their involution, menstruation typically resumes in the absence of exclusive breastfeeding, and resumption of coital activity is generally recommended, provided the woman is clinically recovered [12,13]. During this period, sexual function may be influenced by overlapping biological, hormonal, and psychosocial factors that justify its analysis as a homogeneous and clinically meaningful window of observation. The 11.17-point reduction in total FSFI score (27.81 to 16.64) places this cohort among those with the largest reported decreases in prospective longitudinal studies of postpartum sexual function [1,7,14], and the magnitude exceeds the threshold for clinical relevance established by the original FSFI validation studies [10]. The domain-level analysis reveals a generalized and biologically coherent pattern of dysfunction affecting all six dimensions of the sexual response cycle.

The greatest decline occurred in lubrication (−2.06 points), establishing it as the most severely compromised dimension of postpartum sexual function in our cohort. This finding aligns with the postpartum hormonal milieu, in which low estrogen levels promote vaginal dryness, reduced genital trophism, and impaired vasocongestive response [4,15]. Recent literature has formalized this constellation of symptoms under the construct of postpartum and lactation-related genitourinary syndrome, characterized by vaginal atrophy, dryness, and pain during sexual activity secondary to a hypoestrogenic state [4]. The prominent lubrication deficit observed in our sample, therefore, represents not merely a statistical change but a physiologically coherent postpartum phenotype with direct clinical implications for management.

A particularly informative finding is the near-identical magnitude of decline in arousal (−1.71) and desire (−1.69). This symmetry suggests a coordinated disruption of the early phases of the sexual response cycle, consistent with Basson's circular model in which desire and arousal are interdependent and mutually reinforcing rather than sequential [16]. In the postpartum context, this co-disruption is biologically plausible: elevated prolactin levels suppress hypothalamic dopaminergic activity, reducing desire [4], while concurrent fatigue, sleep deprivation, and emotional adaptation to the maternal role attenuate arousal capacity [7,17]. The fact that both domains decline almost identically rather than independently supports the interpretation that postpartum sexual dysfunction is not a collection of isolated symptoms but a syndromic response to overlapping biological and psychosocial stressors.

The orgasm domain showed a smaller decline (−1.46) yet exhibited the greatest increase in standard deviation (from 0.42 to 0.90). This dispersion indicates substantial inter-individual variability: while a majority experienced complete or near-complete loss of orgasmic capacity, a meaningful subgroup retained moderate function. This heterogeneity is clinically informative, suggesting that orgasmic capacity may depend more on the integrity of upstream domains (lubrication, arousal) than on independent factors. Loss of desire and impaired physiological arousal likely create a cascading effect that culminates in orgasmic dysfunction, an observation consistent with prospective evidence showing that orgasmic function tends to recover later than other domains [1,7].

The pain domain showed a statistically significant deterioration in the postpartum period (−0.95 points; p < 0.001). Since the FSFI scale assigns higher values to less pain, this reduction indicates a meaningful increase in sexual pain during the early postpartum period. The categorical analysis supports this finding: 8.2% of women reported high-frequency pain postpartum, a category entirely absent during pregnancy, while moderate-frequency pain affected more than half of the sample. Although various studies have described associations between mode of delivery and postpartum sexual function, including instrumental delivery, episiotomy, and perineal trauma, evidence remains heterogeneous regarding their direct influence on pain, dyspareunia, and sexual satisfaction [18-20]. In the present study, mode of delivery showed no statistically significant association with postpartum sexual dysfunction after multivariable adjustment (aPR = 1.055; 95% CI: 0.911-1.221; p = 0.479), consistent with evidence indicating that mode of delivery does not exert a significant independent effect on postpartum sexual function [14]. These findings reinforce the necessity for a comprehensive sexual health assessment regardless of the obstetric mode of delivery.

The multivariable analysis identified emotional symptoms (anxiety or sadness) as the strongest predictor of postpartum sexual dysfunction (aPR = 5.06), an association that aligns with longitudinal evidence linking depressive symptoms to impaired sexual function during pregnancy and postpartum [21,22]. The magnitude of this effect, nearly five times the prevalence of dysfunction in women with emotional symptoms, is consistent with the findings of Gokyildiz et al. [23], who reported a significant relationship between depression and impaired sexual function in a multicenter Turkish cohort, and with El-Fakahany and El-Kak [24], who emphasized the centrality of emotional and relational factors in sexual desire and activity during the perinatal period.

The protective association observed for participation in institutional support groups (aPR = 0.63) represents a clinically interesting finding. The 37% reduction in dysfunction prevalence among participants suggests that structured psychosocial spaces may facilitate adaptation to physical and emotional changes, improve partner communication, and buffer the impact of stress on sexual well-being, consistent with evidence showing that women who receive structured guidance report better sexual outcomes [5,6]. However, this finding warrants cautious interpretation. The observational nature of this association precludes causal inference: women who choose to participate in support groups may differ systematically from non-participants in unmeasured characteristics such as baseline emotional resilience or proactive health behaviors, which could act as confounding factors. To establish a true protective effect, a more rigorous design is needed, ideally a randomized controlled trial or quasi-experimental study comparing structurally similar groups with and without a structured support intervention, with pre- and post-intervention FSFI assessments.

Collectively, these findings suggest that postpartum sexual dysfunction transcends purely hormonal or mechanical etiologies and necessitates a comprehensive interpretation within a broader biopsychosocial framework in which biological factors (hypoestrogenism, surgical recovery, fatigue), psychological factors (anxiety, depression, body image distress), and social factors (partner support, access to information, cultural barriers) interact to shape sexual recovery. In this cohort, all participants reported that body image changes negatively affected sexual desire and self-esteem. While the absence of variability in this variable precludes inferential analysis, the result remains clinically meaningful and consistent with evidence linking postpartum body image dissatisfaction with reduced sexual interest and impaired relationship intimacy [25].

Multiparous women showed a higher prevalence of sexual dysfunction compared to primiparous women, although with a modest effect size (aPR = 1.20). International evidence on the role of parity shows heterogeneous results, with some studies suggesting an association with cumulative pelvic floor changes and others finding no significant effect [24,26]. Given the small effect size and the confidence interval close to the null value, this finding should be interpreted with caution and considered a secondary factor within the explanatory model.

The association between higher educational level and increased prevalence of dysfunction (aPR = 1.84) is a counterintuitive finding that warrants reflection. One plausible interpretation is that women with higher education may more readily identify and report dysfunction due to greater awareness of sexual health norms, rather than experiencing higher absolute rates. While some international studies link higher education to better sexual outcomes [7], previous evidence from Spanish and Turkish populations supports the association observed in our cohort, suggesting that specific cultural and contextual determinants shape this relationship [27,28].

Maternal age was not significantly associated with sexual dysfunction, consistent with previous studies indicating that emotional, relational, and contextual factors carry greater weight than demographic variables in postpartum sexual health [7,14,29].

From a clinical perspective, these results support the integration of sexual health into postpartum care as a routine and structured component [13]. Systematic FSFI screening at the 4-8 week postpartum visit should be considered, as this window captures the nadir of sexual function and represents an actionable point of intervention. Mental health screening should be integrated with sexual health assessment, given the strong association between emotional symptoms and dysfunction. The pain domain warrants individualized evaluation regardless of mode of delivery, given that sexual pain emerged as a clinically significant finding in this cohort. Structured support group participation should be facilitated as a low-cost candidate intervention, while recognizing that its protective effect requires confirmation through rigorous interventional studies. Simple measures such as anticipatory guidance, vaginal lubricants, pelvic floor rehabilitation, and psychosocial support may meaningfully improve postpartum recovery and quality of life [4,30].

A particularly salient finding was that none of the participants reported receiving formal sexual health counseling, highlighting a potentially important gap in routine maternal care. Despite the high prevalence of dysfunction documented here, no professional guidance or anticipatory counseling was reported by any participant, which may reflect a broader systemic gap consistent with previous reports of unmet informational needs in postpartum populations [5,6]. This finding highlights the need to systematically incorporate sexual health discussions into routine perinatal care as both a preventive measure and a response to the multifactorial determinants identified in this study.

Strengths and limitations

This study has several strengths: its prospective longitudinal design, the use of a validated multidimensional instrument, assessment at two clinically relevant time points, and the application of Poisson regression with robust variance. Despite these strengths, several limitations must be acknowledged. First, single-center convenience sampling was used, which introduces potential selection bias. Second, restricting follow-up to women who had already resumed sexual activity at 4-8 weeks may underestimate the true prevalence of severe dysfunction, as those experiencing significant pain or profound loss of desire might delay coitarche beyond this window. Third, psychosocial variables (such as partner support and emotional symptoms) were evaluated using self-reported tools rather than validated psychometric scales, introducing potential information bias. Furthermore, cultural reporting bias and social desirability in this Central American cohort may have influenced responses regarding intimate sexual symptoms. Finally, the absence of breastfeeding data is a limitation. These constraints should inform the design of future multicenter and longer-term studies in this regional context.

## Conclusions

The early postpartum period represents a critical window of vulnerability for female sexual health that current maternal care fails to address. Sexual function deteriorates broadly and significantly during this stage, with statistically significant reductions across all six FSFI domains, shaped by the interaction of biological, emotional, and social factors rather than by any single cause. The absence of reported sexual health counseling in this cohort highlights a potentially important gap that warrants attention in routine maternal care. Integrating routine sexual health screening and biopsychosocial support into postpartum care is essential to safeguard maternal well-being, particularly in resource-limited settings where this dimension of health remains invisible.
